# Ubiquitin-specific proteases (USPs) in leukemia: a systematic review

**DOI:** 10.1186/s12885-024-12614-x

**Published:** 2024-07-25

**Authors:** Alireza Zangooie, Shima Tavoosi, Mahan Arabhosseini, Aram Halimi, Helia Zangooie, Amir Hossein Baghsheikhi, Soheila Rahgozar, Mohammad Ahmadvand, Alireza Mosavi Jarrahi, Zahra Salehi

**Affiliations:** 1 Student Research Committee, Birjand University of Medical Sciences, Birjand, Iran; 2 Cellular and Molecular Research Center, Birjand University of Medical Sciences, Birjand, Iran; 3 Department of Cell and Molecular Biology and Microbiology, Faculty of Biological Science and Technology, University of Isfahan, Isfahan, Iran; 4 Non-Communicable Diseases Research Center, Endocrinology and Metabolism Population Sciences Institute, Tehran University of Medical Sciences, Tehran, Iran; 5 Research Center for Social Determinants of Health, Research Institute for Endocrine Sciences, Shahid Beheshti University of Medical Sciences, Tehran, Iran; 6 Department of Epidemiology, School of Public Health and Safety, Shahid Beheshti University of Medical Sciences, Tehran, Iran; 7 Student Research Committee, Mashhad University of Medical Sciences, Mashhad, Iran; 8 Department of Biology, Science and Research Branch, Islamic Azad University, Tehran, Iran; 9 Cell Therapy and Hematopoietic Stem Cell Transplantation Research Center, Research Institute for Oncology, Hematology, and Cell Therapy, Tehran University of Medical Sciences, Tehran, Iran; 10 Cancer Research Centre, Shahid Beheshti University of Medical Sciences, Tehran, Iran; 11 Hematology, Oncology and Stem Cell Transplantation Research Center, Research Institute for Oncology, Hematology and Cell Therapy, Tehran University of Medical Sciences, Tehran, Iran

**Keywords:** Leukemia, Ubiquitin proteasome system, Ubiquitin-specific proteases, Deubiquitinase enzymes

## Abstract

**Background:**

Leukemia, a type of blood cell cancer, is categorized by the type of white blood cells affected (lymphocytes or myeloid cells) and disease progression (acute or chronic). In 2020, it ranked 15th among the most diagnosed cancers and 11th in cancer-related deaths globally, with 474,519 new cases and 311,594 deaths (GLOBOCAN2020). Research into leukemia’s development mechanisms may lead to new treatments. Ubiquitin-specific proteases (USPs), a family of deubiquitinating enzymes, play critical roles in various biological processes, with both tumor-suppressive and oncogenic functions, though a comprehensive understanding is still needed.

**Aim:**

This systematic review aimed to provide a comprehensive review of how Ubiquitin-specific proteases are involved in pathogenesis of different types of leukemia.

**Methods:**

We systematically searched the MEDLINE (via PubMed), Scopus, and Web of Science databases according to the Preferred Reporting Items for Systematic Reviews and Meta-Analyses guidelines (PRISMA) to identify relevant studies focusing on the role of USPs in leukemia. Data from selected articles were extracted, synthesized, and organized to present a coherent overview of the subject matter.

**Results:**

The review highlights the crucial roles of USPs in chromosomal aberrations, cell proliferation, differentiation, apoptosis, cell cycle regulation, DNA repair, and drug resistance. USP activity significantly impacts leukemia progression, inhibition, and chemotherapy sensitivity, suggesting personalized diagnostic and therapeutic approaches. Ubiquitin-specific proteases also regulate gene expression, protein stability, complex formation, histone deubiquitination, and protein repositioning in specific leukemia cell types.

**Conclusion:**

The diagnostic, prognostic, and therapeutic implications associated with ubiquitin-specific proteases (USPs) hold significant promise and the potential to transform leukemia management, ultimately improving patient outcomes.

## Introduction

Leukemia encompasses a diverse spectrum of blood cancers characterized by the uncontrolled proliferation of immature white blood cells. This malignancy is categorized according to both the rapidity of cell division (acute or chronic) and the lineage of the affected cells (myeloid or lymphoid) [1]. The landscape of leukemia subtypes is dominated by myeloid malignancies like acute myeloid leukemia (AML) and chronic myeloid leukemia (CML), while acute lymphoblastic leukemia (ALL) and chronic lymphocytic leukemia (CLL) represent the primary lymphoid forms. Rarer variants, such as those originating from mature B-cell, T-cell, and NK-cell lineages, reveal the diverse spectrum of leukemia [2]. Molecular and cytogenetic assays are foundational tools in leukemia diagnosis, serving key roles in classification, risk stratification, treatment monitoring, and guiding targeted therapies. These multifaceted tests provide crucial insights into genomic aberrations and cellular characteristics, empowering clinicians to tailor treatment strategies for optimal patient outcomes [3].

The ubiquitin proteasome system (UPS), the cell’s central protein degradation machinery, exerts tight control over critical proteins governing cell cycle arrest, apoptosis, and differentiation. In hematological malignancies, dysregulation of the UPS promotes pro-cancerous activities [4]. Irregularities in the various elements comprising the UPS (including E1, E2, E3, DUBs, and proteasome) can play a role in the development of cancer. Specifically, a subset of E3 ubiquitin ligases undergo mutations, overexpression, or deletion in blood-related cancers, causing abnormal buildup or breakdown of their designated targets. Consequently, this disrupted natural breakdown process affects the levels of certain onco-suppressors or oncogenes, thereby influencing the growth, differentiation, and survival of hematopoietic cells [4]. Also, a particular group of enzymes known as deubiquitinating enzymes (DUBs) plays a crucial role by reversing the ubiquitin modification on proteins, thereby preventing their degradation and bolstering their stability. The biggest group of DUBs is represented by the USPs. Irregularities in these enzymes have been observed in various hematological malignancies and cancers, leading to an imbalance between protein breakdown and preservation, resulting in the accumulation of essential proteins involved in diverse biological processes. This imbalance DUBs as viable targets for drug intervention, as their activity can be controlled using small molecule inhibitors, offering promising avenues for therapeutic development in cancer treatment [4–7]. So interest in the USPs as a therapeutic target has increased, leading to the development of small molecules to modulate this pathway.

This systematic review delves into the impact of USP enzymes on the progression of different types of leukemia while also introducing potential therapeutic and diagnostic biomarkers for leukemia. We will illustrate the modulation of USP enzymes, showcasing their impact on leukemia progression, suppression, and altered sensitivity to chemotherapy. This occurs through the regulation of gene expression, manipulation of protein stability, formation of complexes, deubiquitination of histones, and repositioning of other proteins within lymphoid and myeloid leukemia cell types.

## Materials and methods

### Data sources and search strategies

This study adhered to the Preferred Reporting Items for Systematic Reviews and Meta-Analyses guidelines (PRISMA) [8]. We considered articles published in English until July 2023, conducting searches across three databases: PubMed, Web of Science, and Scopus, for eligible studies. The specific search approaches are detailed in Supplementary Table 1. The organization and removal of duplicate articles were handled using EndNote version 20.

### Inclusion and exclusion criteria

In the process of identifying relevant studies, certain inclusion and exclusion criteria were applied. Inclusion criteria included studies that explore the involvement of ubiquitin-specific peptidases in leukemia, published in English, original research articles focusing on human subjects or relevant leukemia cell lines, and those offering insights into the role of ubiquitin-specific peptidases in the pathogenesis, progression, diagnosis, prognosis, or treatment of leukemia. Conversely, exclusion criteria encompassed studies unrelated to the role of ubiquitin-specific peptidases in leukemia, non-English publications, non-original articles such as reviews, conference abstracts, editorials, or letters, animal or in vitro studies not directly applicable to leukemia, and duplicate articles or studies lacking sufficient data.

### Data extraction

Relevant data from included articles are systematically extracted. This includes information such as gene symbol, gene role (e.g., oncogene, Tumor suppressor), targets, and key findings related to the role of ubiquitin-specific peptidases in leukemia.

## Results

**Fig. 1:**
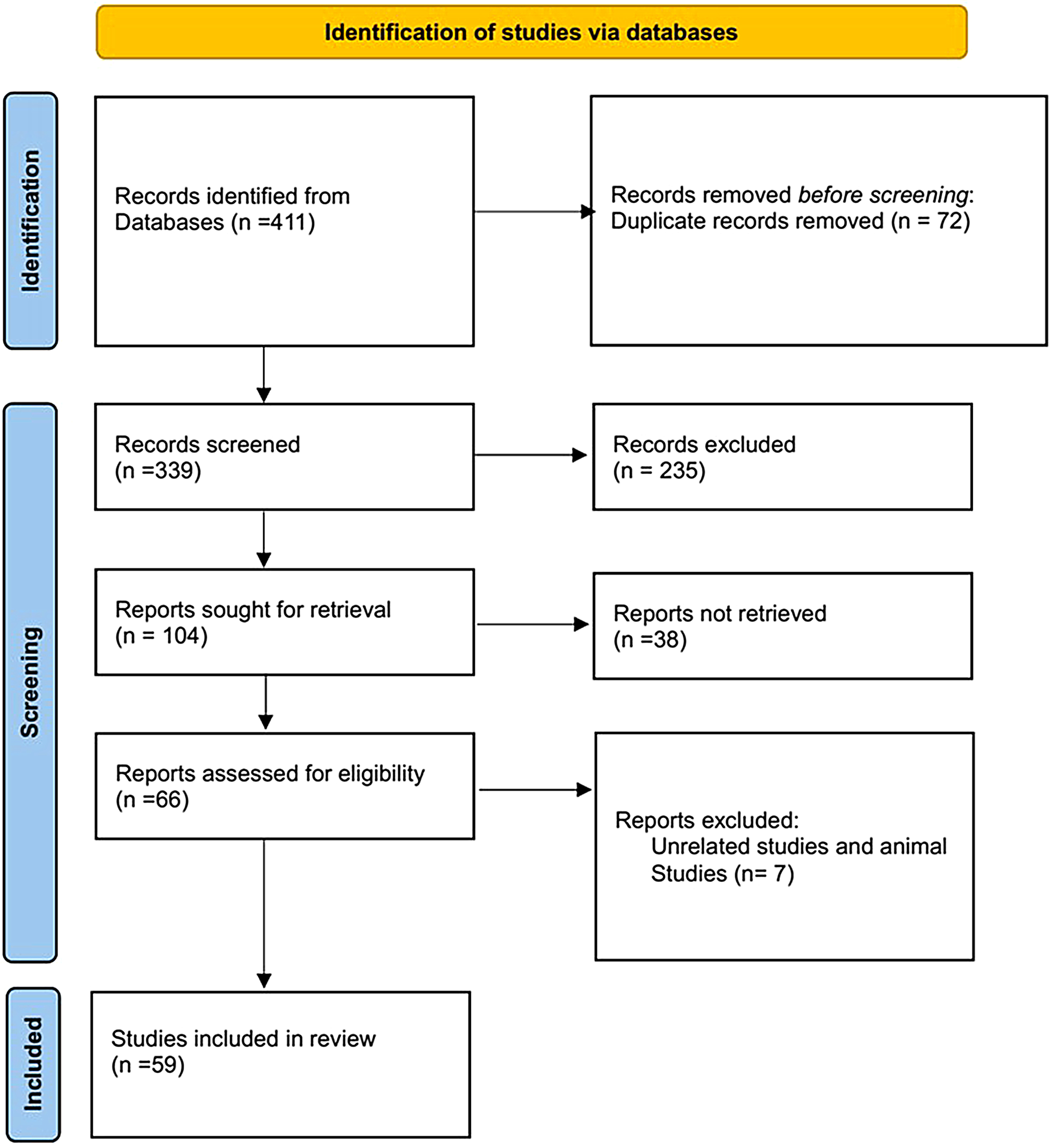
Flowchart of study selection

The article selection process is shown in Fig. 1. Overall, 411 records were identified from the databases. After removing duplicate articles, 339 studies were initially included for screening titles and abstracts; of these 235 were excluded and 104 studies were included for full-text screening. Of these, 45 were excluded because they did not investigate the role of USPs in Leukemia (Fig. 1). Finally, 59 studies were included in the systematic review (Table 1).

**Table 1:** Summary of key findings in studies on the role of ubiquitin-specific peptidases (USPs) in leukemia

No.	Gene symbol	Role	Target	Function and Remarks	Leukemia	References
1	USP42	oncogene	-	RUNX1-USP42 fusion genes induce uncontrolled AML cells growth and impaired their differentiation.	AML	[9–12]
2	USP43	oncogene	-	In mice to have the AML1-ETO fusion, the overexpression of UBP43 in monoblastic M1 AML cells inhibits their maturation into myeloid cells.	AML	[13]
3	USP22	Tumor suppressor	PU.1	USP22 can enhance myeloid differentiation in Kras^G12D/+^ mice by stabilizing the PU.1 protein and decreasing the risk of AML.	AML	[14]
4	USP10	oncogene	FLT3	USP10 can stabilize the FLT3 oncoprotein, leading to an increase in the proliferation of AML cells	AML	[15]
5	USP7	oncogene	CHK1	USP7 via stabilizing CHK1 could enhance the effects of cytarabine, resulting in the killing of AML cell lines and making them more responsive to chemotherapy.	AML	[16]
6	USP2 and 21	oncogene	-	Disulfiram and 6-thioguanine (6TG) via exhibiting a synergistic inhibitory effect on USP2 and 21 enforce their cytotoxicity on AML cells.	AML	[17]
7	USP22	oncogene	Cyclin D1	USP22 could stabilize Cyclin D1 through its interaction with RNF220 so it can increase AML cells proliferation.	AML	[18]
8	USP14	oncogene	-	b-AP15 molecule via inhibiting UCHL5 and USP14 can restrain organ infiltration in AML cases that are unresponsive to TP53 status and exhibit overexpression of the anti-apoptotic protein BCL2.	AML	[19]
9	USP30	oncogene	-	lncRNA USP30-AS1, by cis-regulating the USP30 gene, can progress AML.	AML	[20]
10	USP15	Tumor suppressor	KEAP1-NRF2	Knockdown of USP15 can activate an antioxidant response via upregulating the KEAP1-NRF2 AML, substantially negatively impacting the function and survival of leukemic progenitor cells.	AML	[21]
11	USP7	oncogene	mTOR	USP7 may enhance AML cell growth by activating mTOR.	AML	[22]
12	USP3	oncogene	-	USP3 by removing the H2AK119ub may counteract the effects of the PRC1 complex, potentially promoting leukemia cell differentiation.	AML	[23]
13	USP18	oncogene	PLK2	USP18 suppression could induce cancer cell GSDME-dependent pyroptosis and suppress leukemogenesis via upregulating PLK2	AML	[24]
14	USP37	oncogene	PLZF/RARA	USP37 via stabilizing PLZF/RARA fusion protein through the PLZF portion could promote APL cells formation.	APL	[25]
15	USP43/USP18	oncogene	PLZF/RARA	UBP43/USP18 reduce in apoptosis in APL cells via stabilizing PLZF/RARA fusion protein.	APL	[26]
16	USP48	Tumor suppressor	-	MiR-301a-3p could inhibit granulocytic differentiation that ATRA induces via sponging USP48.	APL	[27]
17	USP39	oncogene	-	USP39 by reducing the expression of Caspase 8 and IRF1 while increasing the levels of Sp1 reduce rate of APL, T-ALL, and CML cell lines apoptosis.	APL, T-ALL, CML	[28]
18	USP7	oncogene	PML	The EBV protein EBNA1 by forming a ternary complex with USP7 and CK2 resulting in the degradation of PML proteins. This disruption of PML-NBs leads to impaired DNA repair and increased APL cell survival.	APL	[29]
19	HAUSP	oncogene	PTEN	PML NBs regulate PTEN localization by countering overexpression of PTEN-deubiquitinating enzyme called HAUSP, which can lead to PTEN nuclear exclusion. The nuclear exclusion of the PTEN tumor suppressor is linked to cancer progression.	APL	[30]
20	HAUSP	Tumor suppressor	PTEN	The mutated NPM1 (NPMc+) prevents HAUSP from deubiquitinating PTEN. As a result, PTEN remains in the cytoplasm, and is subsequently degraded. This series of events affects the cellular localization and stability of PTEN, which is significant in the context of AML and its progression.	AML	[31]
21	USP18	oncogene	-	USP18 is upregulated in APL cells exposed to IC50 concentrations VOCs that it participates in downregulation of immune response and IFN-related genes through the JAK-STAT pathway via IFN against APL cells	APL	[32]
22	USP1	oncogene	Bcr-Abl	When USP1 protein activity was suppressed it led to a decrease in the amount of the Bcr-Abl oncoprotein in CML cells.	CML	[33]
23	USP10	oncogene	SKP2	USP10 stabilizes SKP2 and therefore activates the SKP2/Bcr-Abl signaling pathway, potentially leading to increased proliferation of both imatinib-sensitive and imatinib-resistant CML cells.	CML	[34]
24	USP25	oncogene	Bcr-Abl	USP25 could decrease CML cell’s senitivity to TKIs via stabilizing BCR-ABL protein in cells carrying the Philadelphia chromosome (Ph).	CML	[35]
25	USP7	oncogene	RNF6	USP7 associates with RNF6 and disrupts its K48-linked polyubiquitination process, which ultimately stops its degradation. This action results in decrease cell death in multiple myeloma (MM) and CML cells.	CML, MM	[36]
26	USP1	oncogene	Bcr-Abl	USP1 protein with the PH domain of the Bcr-Abl protein can enhance the accumulation of CML cells and advance the progression of CML.	CML	[37]
27	USP7	oncogene	Bcr-Abl	ART effectively disrupts the interaction between USP7 and BCR-ABL, leading to the degradation of BCR-ABL and ultimately causing the death of CML cells.	CML	[38]
28	USP47	oncogene	YBX1	USP47 promotes AML cell proliferation, enhances resistance to imatinib, and eliminates leukemia progenitor cells in CML by stabilizing YBX1.	CML	[39]
29	USP43	oncogene	Bcr-Abl	Removal of UBP43 (also known as USP18) resulted in a reduction in the development of BCR-ABL-induced leukemia. This reduction was achieved by controlling the signaling of the Type 1 interferon (IFN-α/β) receptor.This process induce apoptosis of CML cells.	CML	[40]
30	USP15	oncogene	FUS	USP15 increases the stability of FUS), a factor that involved in DNA repair. This interaction may play a role in connecting USP15 to the DNA damage response (DDR). So when USP15 is reduced in murine precursor cells and leukemia cells, it decreases their ability to proliferate and heightens the level of genotoxicity.	CML	[41]
31	USP1	oncogene	ID1	Inhibition of USP1 lead to the breakdown of ID1 and are harmful to leukemia cells.	CML	[42]
32	HAUSP	oncogene	PTEN	In CML, BCR-ABL a fusion protein via the increasing de-ubiquitinating activity of HAUSP toward PTEN could interfere with PTEN by promoting its removal from the cell nucleus. Hence, BCR-ABL promotes cancer in CML by preventing PTEN from functioning properly in the cell nucleus.	CML	[43, 44]
33	USP14 and UCHL5	oncogene	Bcr-Abl	NiPT triggers apoptosis and activates caspase by hindering the ubiquitin-proteasome system. It does this by specifically targeting the 19 S proteasome-associated deubiquitinases (USP14 and UCHL5). As a result, NiPT induces apoptosis in CML cells that are resistant to imatinib, doing so through both Bcr/Abl-independent and Bcr/Abl-dependent mechanisms.	CML	[45]
34	USP6	oncogene	GLS1	USP6 is associated with an increase in resistance to imatinib by stabilizing the GLS1 protein. But hucMSC exosomes enhance imatinib-induced apoptosis in CML cells by regulating the miR-145a-5p/USP6/GLS1 axis.	CML	[46]
35	USP15	oncogene	Caspase-6	Although USP15 increases the level of caspase-6 in CML cells by deubiquitinating it, the activation of the STAT5A/miR-202-5p/USP15/Caspase-6 signaling pathway can lead to imatinib resistance and ameliorate apoptosis in CML cells.	CML	[47]
36	HAUSP	oncogene	P53	p190 BCR-ABL fusion protein, through its interaction with the HAUSP, could induce the destabilization and subsequent downregulation of the p53 protein.	ALL	[48]
37	USP15	oncogene	-	USP15 plays crucial roles in the transformation of hematopoietic cells into AML or T-ALL cells induced by BCR-FGFR.	AML, T-ALL	[49]
38	USP7	Tumor suppressor	SRSF6	USP7 via destabilizing SRSF6 leads to alterations in exon skipping patterns and inhibits the growth of T-ALL.	T-ALL	[50]
39	USP15	oncogene	MDM2 and KEAP1	TIFAB-USP15 complex through the deubiquitination of MDM2 and KEAP1, may lead to a reduction in p53 expression. This, in turn, could enhance the activity of leukemic cells and contribute to the progression of leukemia.	ALL	[51]
40	USP1	oncogene	ID1/AKT	USP1 promotes B-ALL progression through the ID1/AKT signaling pathway.	B-ALL	[52]
41	USP1	oncogene	Aurora B	USP1 is overexpressed, it decreases the beneficial effects of an Aurora B inhibitor by interacting with Aurora B. Then it increase T-ALL resistance to dexamethasone which could decrease cell apoptosis	T-ALL	[53]
42	USP7 and 11	oncogene	LCK	USP11 collaborates with USP7 to remove ubiquitin molecules from the oncogenic protein LCK. This action boosts LCK’s activity, promoting the progression of an ALL while impairing normal blood cell formation and reducing sensitivity to GC.	ALL	[54]
43	USP7	Tumor suppressor	-	In T-ALL, USP7 forms a co-regulatory complex with HEB, TAL1 and E2A then inhibiting T-ALL cell growth.	T-ALL	[55]
44	USP7	Tumor suppressor	NOTCH1	By reducing the degradation of NOTCH1, USP7 can mitigate apoptosis in T-ALL cells.	T-ALL	[56]
45	USP7	oncogene	NOTCH1	USP7 plays a role by stabilizing NOTCH1 and subsequently activating the NOTCH1/JMJD3 pathway. This activation, in turn, increase the growth of T-ALL cells.	T-ALL	[57]
46	USP20	oncogene	TRAF6 and Tax	USP20, by promoting the stability of TRAF6 and Tax, contributes to the activation of NF-κB, including its activation induced by IL-1β.	T-ALL	[58]
47	USP9x	Tumor suppressor	Mcl-1	Ionizing radiation activates USP9x, leading to increased deubiquitination of Mcl-1 in radioresistant T-ALL cells. This sensitizes them to apoptosis.	T-ALL	[59]
48	USP24	Tumor suppressor	Mcl-1	WP1130 treatment reduced the survival of T-ALL cells, prompting apoptosis, in vivo and in vitro. This effect was achieved by increasing the disruption of the mitochondrial transmembrane potential through the USP24-Mcl-1 pathway.	T-ALL	[60]
49	USP10	Tumor suppressor	-	USP10-containing SGs help reduce the production of reactive oxygen species (ROS) and inhibit apoptosis triggered by ROS. However, the interaction between the Tax oncoprotein of HTLV-1 and USP10 has the opposite this effect and could copotentially enhance apoptosis in T-ALL cells.	T-ALL	[61]
50	USP2	oncogene	-	Based on enzyme kinetics and X-ray crystallography, 6TG that is a long-standing medication currently employed in the treatment of leukemia, operates as a noncompetitive inhibitor with a slow-binding mechanism against USP2.	ALL	[62]
51	USP7	oncogene	-	Inhibiting USP7 in ALL cells could lead to a reduction in MDM2 expression and an increase in p21 and p53 expression.	T-ALL	[63]
53	USP33	oncogene	-	USP33 and endomucin (EMCN), which were found to be overexpressed in B-ALL in comparison to their expression in T-ALL.	B-ALL and T-ALL	[64]
54	USP44	oncogene	-	Elevated USP44 expression is found in some T-ALL cells.	T-ALL	[65]
55	USP7	oncogene	-	Inhibiting USP7 disrupts HRR, causing a significant buildup of DNA damage and leading to CLL cell apoptosis, even in cases where ATM and p53 are dysfunctional. Additionally, inhibiting USP7 makes p53-defective and chemotherapy-resistant CLL cells more responsive to clinically relevant doses of chemotherapy agents that induce HRR.	CLL	[66]
56	USP7	oncogene	-	USP7 reduces apoptosis in CLL cells. Additionally miR-181b and miR-338-3p, by sponging USP7 mRNA, can reduce its protein levels. In contrast, CK2 can trigger PTEN’s exclusion from the cell nucleus by activating USP7.	CLL	[67]

### USPs in myeloid leukemia

#### AML

Acute myeloid leukemia (AML) is a diverse malignant condition associated with a poor prognosis. Ubiquitination, a significant post-translational modification (PTM), is crucial for controlling diverse cellular processes and influencing cell fate. Despite initial observations, the specific involvement of ubiquitination in the development and treatment of AML is not yet fully understood [68]. Chromosomal abnormalities, particularly fusion genes like RUNX1-USP42, play a significant role in the AML. In AML, the emergence of the RUNX1-USP42 fusion gene is primarily due to the translocation between chromosomes 7 and 21. A limited number of AML patients exhibiting t(7;21)(p22;q22) have been documented, often accompanied by additional chromosomal irregularities, particularly deletions of the long arm of chromosome 5 (5q). The co-occurrence of t(7;21) and the loss of the 5q segment seems to be a non-random discovery in AML patients. These genetic abnormalities disrupt the normal functioning of cells, leading to uncontrolled cell growth and impaired differentiation [9–12]. A comprehensive understanding of these abnormalities is essential to diagnose and manage AML effectively. Furthermore, Gigue`re and coworkers have detected the existence of consensus sequences of Topoisomerase II and microhomologies in proximity to the breakpoint junctions of the recurring t(7;21)(p22;q22) translocation in AML [11].

The AML1-ETO fusion, associated with a specific chromosomal abnormality t(8;21)(q22;q22), is a common genetic anomaly in AML, occurring in around 15% of adult AML patients. A study by Schwer and colleagues found that in mice to have the AML1-ETO fusion, the overexpression of UBP43 in monoblastic M1 AML cells inhibits their maturation into myeloid cells [13].

NRAS or KRAS mutations detected in approximately 20–40% of myeloid malignancies and 10–15% of acute lymphoblastic leukemia cases. When the most common KRAS mutation, G12D, expressed in mouse hematopoietic cells, it leads to a lethal condition known as myeloproliferative neoplasm (MPN). Research has shown that USP22 can enhance myeloid differentiation in Kras^G12D/+^ mice by stabilizing the PU.1 protein and decreasing the risk of AML [14].

Around 30% of individuals diagnosed with AML carry mutations that activate the FLT3 gene, which typically regulates hematopoiesis. In addition, the most prevalent FLT3 mutation in AML involves internal tandem duplications (ITD) in the juxtamembrane domain. This mutation occurs in about 20–25% of AML patients and is linked to significantly reduced survival rates. Weisberg and their team demonstrated that USP10 can stabilize the FLT3 oncoprotein, leading to an increase in the proliferation of AML cells [15].

In FLT3-ITD-positive acute myeloid leukemia, suppressing CHK1 activity reduces cell proliferation [69]. USP7 plays a role in interacting with and regulating CHK1 protein levels and functions within AML. When USP7 is inhibited, it enhances the effects of cytarabine, resulting in the killing of AML cell lines and making them more responsive to chemotherapy [16].

HC Lin and co-workers discovered that when disulfiram and 6-thioguanine (6TG) utilized in AML therapy, they exhibit a synergistic inhibitory effect on USP2 and 21. This suggests that disulfiram might target the zinc finger domain, as 6TG directly binds to the catalytic cysteines [17].

Ring finger proteins have a distinctive motif and play various roles in biological processes. In the context of cancer, these genes often expressed in abnormal ways. In the case of AML, Pan et al. discovered that the expression of Ring finger protein 220 (RNF220) was linked to negative outcomes, as it was inversely correlated with disease-free survival (DFS) and overall survival (OS) in AML patients. RNF220 stimulates the growth of leukemia cells by diminishing the breakdown of Cyclin D1 protein. Cyclin D1 known to facilitate tumor cell proliferation and push the cell cycle from the G1 phase to the S phase. RNF220 stabilizes Cyclin D1 through its interaction with USP22 [18].

b-AP15, a small molecule, has been identified as a novel type of proteasome inhibitor. It effectively hinders the functions of two deubiquitinase enzymes associated with the 19 S regulatory particle: ubiquitin C-terminal hydrolase 5 (UCHL5) and USP14. Notably, b-AP15 can restrain organ infiltration in AML cases that are unresponsive to TP53 status and exhibit overexpression of the anti-apoptotic protein BCL2 [19].

Zhou and colleagues have uncovered that the long non-coding RNA (lncRNA) USP30-AS1, by cis-regulating the USP30 gene, can advance AML. Additionally, it can influence ANKRD13A, possibly causing the movement of HLA-I protein from the cell membrane to the cytoplasm, which may contribute to immune evasion by AML cells [20].

Deletion or knockdown of USP15 can activate an antioxidant response via upregulating the KEAP1-NRF2 AML in humans and mice, substantially negatively impacting the function and survival of leukemic progenitor cells [21].

PRC2 is a protein complex that includes EZH2, SUZ12, and EED. It adds three methyl groups to histone H3 at lysine 27 (H3K27me3), leading to gene repression. EZH2, the catalytic subunit of PRC2, is often upregulated in cancer, causing the silencing of tumor suppressor genes, DNA damage response genes, and mTOR pathway regulators. Yamini M. Ohol demonstrated that inhibition of USP7 could increase the expression of genes typically silenced by PRC2, potentially by stabilizing or activating PRC2 or its components. Furthermore, EZH2 silences the mTOR-inhibitory proteins FKBP11 and RGS16. In addition, inhibition of USP7, in combination with inhibition of PIM kinase and PI3K, which activate mTOR, synergistically reduce cell proliferation and promote apoptosis in AML cells. This suggests that USP7 may enhance AML cell growth by activating mTOR [22]. The PRC1 complex is responsible for adding ubiquitin molecules to histone H2A at a specific site, lysine 119 (H2AK119ub), a process facilitated by the enzyme Ring1B, which possesses E3 ligase activity. This modification is associated with the development of leukemia. When one of the subunits of PRC1, Cbx7, is overexpressed in hematopoietic and progenitor cells, it promotes leukemia. Conversely, the loss of PRC1 activity induces the differentiation of leukemia cells through its enzymatic actions on H2AK119ub. In a related study by Chae et al., it was observed that the treatment of AML cell lines with TPA, which induces maturation in these cells, led to an increase in USP3 levels in AML cell lines. Furthermore, it is suggested that USP3 may counteract the effects of the PRC1 complex, potentially promoting leukemia cell differentiation, including in cases of leukemia, by removing the H2AK119ub modification [23].

**Fig. 2:**
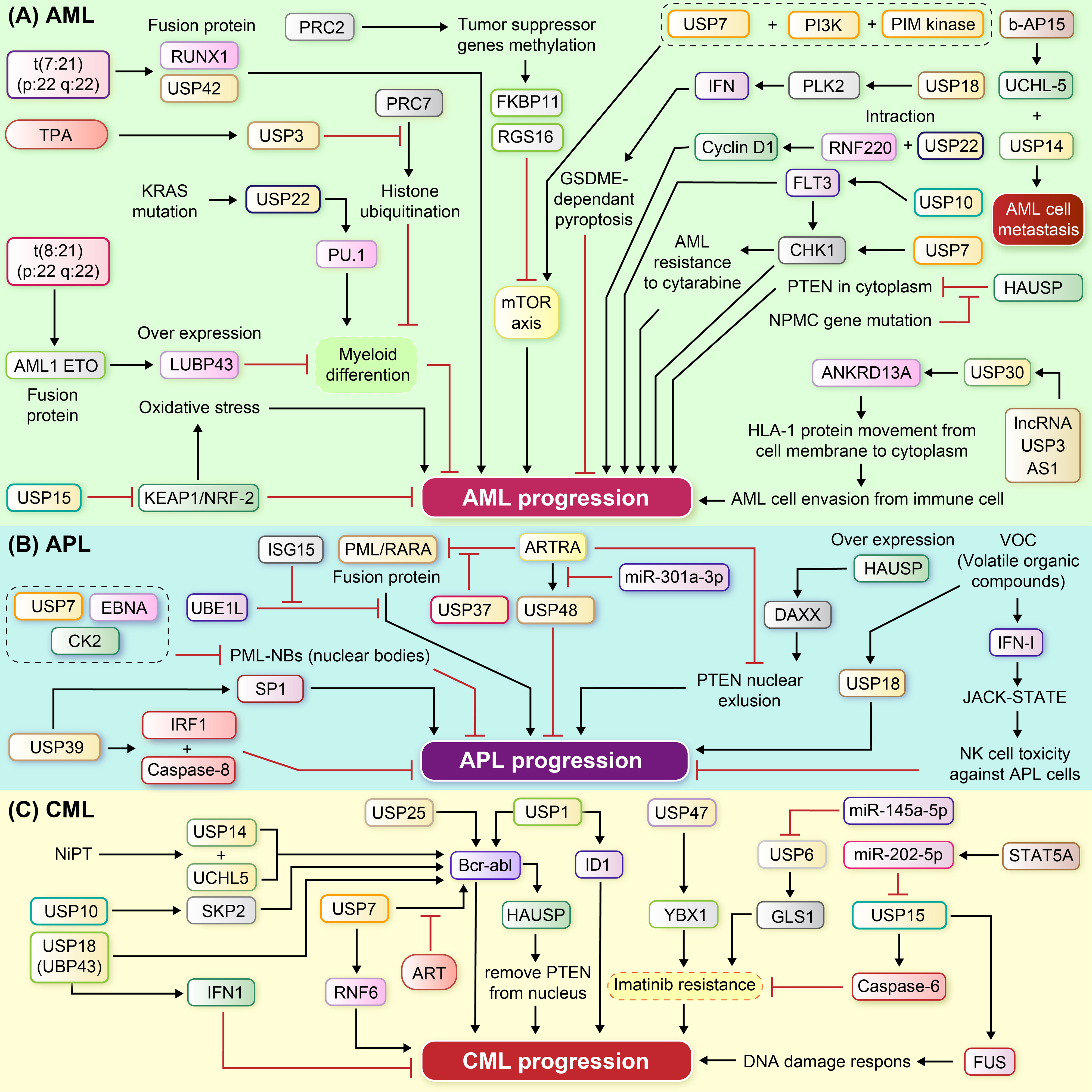
Signaling pathway of the USP enzyme family that regulate myeloid leukemia. (**A**) Critical chromosomal abnormalities, fusion genes, and mutations, such as RUNX1-USP42, AML1-ETO, FLT3-ITD, and KRAS, play pivotal roles in the pathogenesis of AML. Additionally, ubiquitination and stabilization of oncoproteins such as FLT3, CHK1, Cyclin D1, mTOR, and PLK2, as well as tumor suppressor proteins PU.1 and KEAP1-NRF2, play a role in the regulation of AML. Several potential therapeutic targets, including USP22, 10, and 7, and b-AP15, have proven effective for the management of AML. Moreover, the involvement of long non-coding RNA (lncRNA) and the modulation of PRC1 and PRC2 complexes have also been implicated in contributing to the pathogenesis of AML. (**B**) Targeted therapies, such as arsenic trioxide and ATRA treatments specifically designed for PML/RARA fusions by cooperating with USP may advance APL progression. The progression of APL could be influenced by miR-301a-3p, which sponges USP4 8and 39, which stabilizes SP1. Further investigation into the role of PML-NBs, the EBV protein EBNA1 forming a triple protein complex, and HAUSP affecting the cellular location of PTEN, can shed light on their impact on APL progression. (**C**) Various USPs, including USP25, 1, 7, 14, 10, and 18, as well as HAUSP, exert influence on CML progression through interactions with BCR-abl. Potential therapeutic targets such as USP47, USP15, and NiPT, in conjunction with key components like STAT5, miR-202-5p, and the GLS1/USP6 axis, contribute to chemoresistance in CML cells

Type I interferon (IFN) is well known to play important roles in different aspects of immune responses, including modulating immunogenic cell death (ICD) in anti-tumor action. Arimoto et al. demonstrated that reduced USP18 expression in murine leukemia cells alters the IFN-stimulated genes (ISGs) landscape and results in loss of leukemia cells, including cells with features of niche resident LSCs. USP18 suppression could induce cancer cell GSDME-dependent pyroptosis and suppress leukemogenesis via upregulating PLK2, an atypical ISG [24] (Fig. 2A).

### APL

Acute promyelocytic leukemia (APL) constitutes approximately 10–15% of the total cases of AML [70] characterized by genetic rearrangements involving the retinoic acid receptor, alpha (RARA) gene, and either promyelocytic leukemia (PML) or promyelocytic leukemia zinc finger (PLZF) gene, in APL cases with PML/RARA fusions, treatments with arsenic trioxide and all-trans retinoic acid (ATRA) target the fusion protein for degradation through the proteasome, leading to cellular differentiation and clinical remission. On the other hand, APL cells with PLZF/RARA fusion proteins are often resistant to these treatments, resulting in a poor prognosis for affected patients. The research shows that USP37 interacts with PLZF/RARA through the PLZF portion, maintaining stable levels of PLZF/RARA and increasing its ability to suppress target genes and promote cell transformation [25].

Guo and coworkers revealed that when ATRA treatment is applied to APL cells, it causes the degradation of the PML/RARα protein, which is produced due to a chromosomal translocation. The enzyme ubiquitin-activating enzyme-E1-like (UBE1L), resembling E1 ubiquitin-activating enzyme, interacts with the interferon-stimulated gene 15 (ISG15). ISG15, in turn, binds to and inhibits the PML/RARα protein. The enzyme UBP43/USP18 plays a role in removing ISG15 from proteins it is attached to, potentially leading to the stabilization of the PML domain within PML/RARα and a reduction in apoptosis in APL cells [26].

In acute APL, abnormal promyelocytes accumulate in the bone marrow, preventing their maturation into mature granulocytes. ATRA treatment is essential as it induces the transformation of these abnormal cells into mature granulocytes, a critical step in APL therapy. In a study by LI et al., it was shown that the protein USP48 promotes granulocytic differentiation that ATRA induces, while miR-301a-3p inhibits this process by binding to and reducing USP48 translation [27]. Liu and colleagues revealed that USP39, by reducing the expression of Caspase 8 and IRF1 while increasing the levels of Sp1, could control the cell cycle of APL, T-ALL, and CML cell lines and reduce their rate of apoptosis [28].

PML-nuclear bodies (PML-NBs) are involved in various genome maintenance pathways, including the DNA damage response, DNA repair, telomere maintenance, and p53-associated apoptosis [71].

Latent Epstein-Barr virus (EBV) infection is a significant contributor to several cancers, such as nasopharyngeal carcinoma (NPC). In the context of NPC, a study by Sivachandran et al. revealed that the EBV protein EBNA1, found in the nucleus of NPC cells, disrupts PML-NBs. It accomplishes this by forming a ternary complex with USP7 and CK2, resulting in the degradation of PML proteins. This disruption of PML-NBs leads to impaired DNA repair and increased cell survival, potentially contributing to the development of cancer. Remarkably, the study shows that USP7, which is typically associated with stabilizing specific proteins by removing ubiquitin from them, promotes the degradation of PML proteins in this context. Interestingly, this degradation of PML proteins by USP7 does not involve USP7’s usual catalytic activity [29].

The nuclear exclusion of the PTEN tumor suppressor is linked to cancer progression. This aberrant localization is governed by PTEN’s ubiquitination at specific lysine residues in the nucleus. Song et al. revealed that functional PML NBs regulate PTEN localization by countering overexpression of PTEN-deubiquitinating enzyme called HAUSP, which can lead to PTEN nuclear exclusion. In addition, PML antagonizes HAUSP’s activity on PTEN through an adaptor protein, DAXX. In APL cells, where the PML function is compromised due to the presence of the PML-RARα fusion oncoprotein, PTEN is mislocalized. However, treatment with ATRA and arsenic trioxide causing PML-RARα degradation restores nuclear PTEN [30]. Therefore, this study and one other that told in AML section revealed that HAUSP’s normal expression unlike its overexpression has a negative effect against PTEN and remains it in the nucleus.

Approximately one-third of AML cases involve mutations in exon 12 of the Nucleophosmin (NPM1) gene. NPM1 mutations are common in AML and often result in NPM1 being located in the cytoplasm in the mutated form of AML (NPMc + AML). In the nucleus, NPM1 normally opposes the HAUSP-mediated deubiquitination process, which plays a role in regulating PTEN. This opposition promotes the movement of PTEN from the nucleus to the cytoplasm. However, in the cytoplasm, the mutated NPM1 (NPMc+) prevents HAUSP from deubiquitinating PTEN. As a result, PTEN remains in the cytoplasm, where it undergoes polyubiquitination and is subsequently degraded. This series of events affects the cellular localization and instability of PTEN, which is significant in the context of AML and its progression [31].

Type I IFN enhances natural killer cell cytotoxicity and apoptosis. suggests that USP18 is upregulated in APL cells exposed to IC50 concentrations of volatile organic compounds (VOCs) and that it participates in downregulation of immune response and IFN-related genes through the JAK-STAT pathway via IFN against APL cells [32] (Fig. 2B).

#### CML

Chronic myeloid leukemia (CML) is a myeloproliferative disorder affecting hematopoietic stem cells. The exploration of CML’s molecular biology commenced with the identification of the Philadelphia chromosome. This genomic anomaly arises from a t(9;22) translocation, leading to the formation of a BCR-ABL1 fusion gene [72]. USP1 supression led to a decrease in the amount of the Bcr-Abl oncoprotein in CML cells [33].

Over time, resistance to TKIs (tyrosine kinase inhibitors) develops in patients with advanced CML, making these drugs less effective. Researchers are studying the increased expression or activation of the Bcr-Abl protein, which is thought to play a role in the advanced phase of CML. S-phase kinase-associated protein 2 (SKP2) acts as an activator of Bcr-Abl by promoting its K63-linked ubiquitination. USP10 stabilizes SKP2 and therefore activates the SKP2/Bcr-Abl signaling pathway, potentially leading to increased proliferation of both imatinib-sensitive and imatinib-resistant CML cells [34].

Shibata and colleagues have demonstrated that USP25 could decrease CML cell’s senitivity to TKIs via stabilizing BCR-ABL protein in cells carrying the Philadelphia chromosome (Ph) [35].

The zinc finger ubiquitin ligase RNF6 has been suggested as a possible focus for treatment in various cancer types. It can degrade itself through auto-ubiquitination, particularly via its RING domain. Zhuang et al. demonstrate that USP7 associates with RNF6 and disrupts its K48-linked polyubiquitination process, which ultimately stops its degradation. This action results in decrease cell death in multiple myeloma (MM) and CML cells [36].

Antonenko and their colleagues have shown that the nuclear co-localization of the USP1 protein with the PH domain of the Bcr-Abl protein can enhance the accumulation of CML cells and advance the progression of CML [37].

USP7 plays a significant role in enhancing the stability of the BCR-ABL fusion protein. The antimalarial drug artesunate (ART) effectively disrupts the interaction between USP7 and BCR-ABL, leading to the degradation of BCR-ABL and ultimately causing the death of CML cells [38].

The increase in USP47 due to BCR-ABL translocation is facilitated through the activation of the RAS/ERK and STAT5 signaling pathways in CML. Additionally, USP47 promotes AML cell proliferation, enhances resistance to imatinib, and eliminates leukemia progenitor cells in CML by stabilizing Y-box-binding protein 1 (YBX1), a vital regulator of cell survival observed in multiple solid tumors and CML [39].

Yan and colleagues demonstrated that the removal of UBP43 (also known as USP18) resulted in a reduction in the development of BCR-ABL-induced leukemia. This reduction was achieved by controlling the signaling of the Type 1 interferon (IFN-α/β) receptor. Additionally, the absence of UBP43 increased the effectiveness of Type 1 interferon in inducing apoptosis of CML cells [40].

Van den Berk’s research uncovered that USP15 exhibits elevated levels of expression in human hematopoietic tissues and leukemia. When USP15 is reduced in murine precursor cells and leukemia cells, it decreases their ability to proliferate in vitro and heightens the level of genotoxicity. Furthermore, in CML cells, USP15 interacts with and increases the stability of FUS (fused in sarcoma), a well-known factor involved in DNA repair. This interaction may play a role in connecting USP15 to the DNA damage response (DDR) [41].

The transcription factor, Inhibitor of DNA-binding-1 (ID1), plays a crucial role in the growth and advancement of various cancer forms, such as leukemia. Mistry and colleagues have demonstrated that newly developed small-molecule inhibitors of USP1 lead to the breakdown of ID1 and are harmful to leukemia cells [42].

Morotti and coworkers found that in CML cells, HAUSP is present in both the cytosol and the nucleus, with a significant amount in NBs, while normal bone marrow cells primarily have HAUSP in the nucleus. The researchers hypothesized that the cytosolic protein BCR-ABL phosphorylates HAUSP in the cytoplasm, causing it to move into the nucleus then PTEN and HAUSP have been demonstrated to create a compartment alongside PML within the NBs then it could regulate the de-ubiquitination of PTEN in NBs [43]. Another study found that the protein PTEN, which acts as a tumor suppressor, plays a critical role in CML. However, in CML, BCR-ABL a fusion protein via the increasing de-ubiquitinating activity of HAUSP toward PTEN could interfere with PTEN by promoting its removal from the cell nucleus. Hence, BCR-ABL promotes cancer in CML by preventing PTEN from functioning properly in the cell nucleus. Interestingly, this PTEN relocation doesn’t happen in a specific subset of leukemia stem cells because they have a high level of another protein called PML, which prevents HAUSP from affecting PTEN [44].

Lan et al. found that nickel pyrithione (NiPT) triggers apoptosis and activates caspase by hindering the ubiquitin-proteasome system. It does this by specifically targeting the 19 S proteasome-associated deubiquitinases (USP14 and UCHL5). Importantly, NiPT’s actions on the proteasome system don’t affect the 20 S proteasome. Furthermore, NiPT reduces the levels of Bcr-Abl proteins, leading to a decrease in Bcr-Abl transcription and the cleavage of Bcr-Abl protein. As a result, NiPT induces apoptosis in CML cells that are resistant to imatinib, doing so through both Bcr/Abl-independent and Bcr/Abl-dependent mechanisms [45].

An increasing amount of evidence suggests that GLS1 plays a role in decreasing sensitivity to chemotherapy. USP6 is associated with an increase in resistance to imatinib by stabilizing the GLS1 protein. However, the introduction of exosomes from human umbilical cord mesenchymal stem cells (hucMSC) promotes cell apoptosis induced by imatinib. This effect is mediated through the miR-145a-5p/USP6 pathway. In summary, hucMSC exosomes enhance imatinib-induced apoptosis in CML cells by regulating the miR-145a-5p/USP6/GLS1 axis [46].

The involvement of STAT5 is crucial in BCR-ABL alteration of hematopoietic cells caused by. In CML cells, STAT5A is upregulated, and it directly activates the transcription of miR-202-5p by binding to the pre-miR-202 promoter. Additionally, USP15 increases the level of caspase-6 in CML cells by deubiquitinating it. MiR-202-5p, by targeting USP15, reduces its protein level. Consequently, the activation of the STAT5A/miR-202-5p/USP15/Caspase-6 signaling pathway can lead to imatinib resistance and ameliorate apoptosis in CML cells [47] (Fig. 2C).

### USPs in lymphoid leukemia

#### ALL

Acute lymphoblastic leukemia (ALL) encompasses a spectrum of malignancies in B/T-precursor-stage lymphoid cells, originating from genetic changes hindering lymphoid differentiation and promoting abnormal cell proliferation and survival. Advances in next-generation sequencing (NGS) have unveiled novel mutations impacting normal lymphopoiesis, emphasizing the role of cooperating mutations and epigenetic alterations [70]. Philadelphia chromosome-positive (Ph+) ALL with the p190 BCR-ABL variant is characterized by its association with the Philadelphia chromosome. In their study, Carrà and colleagues have shown that the p190 BCR-ABL fusion protein, through its interaction with the deubiquitinase known as herpesvirus-associated ubiquitin-specific protease (HAUSP), can induce the destabilization and subsequent downregulation of the p53 protein [48].

The translocation of Fibroblast Growth Factor Receptors (FGFRs) can result in abnormal cell growth and cancer. Specifically, the BCR-FGFR1 fusion protein, formed through chromosomal translocation t(8;22)(p11;q11), combines the Breakpoint Cluster Region (BCR) with Fibroblast Growth Factor Receptor 1 (FGFR1). This fusion protein is a major driver of 8p11 myeloproliferative syndrome, also known as stem cell leukemia/lymphoma, which can progress into AML or T-ALL. Additionally, several proteins, including, GAB1, ECSIT, PTPN11, USP15, and GPR89 play crucial roles in the transformation of hematopoietic cells induced by BCR-FGFR1 [49].

Splicing alterations are a common occurrence in diseases, particularly cancer. Zhou and coworkers reveal significant changes in the skipping of exons in diseases that affect proteasomal subunits, cell cycle regulators, and RNA-related functions. Further, they provide evidence that serine/arginine-rich splicing factors (SRSF), which regulate exon skipping, play a crucial role in the survival of T-ALL cells. Furthermore, they demonstrated that Inhibiting USP7 via destabilizing SRSF6 leads to alterations in exon skipping patterns and inhibits the growth of T-ALL [50].

Niederkorn and colleagues suggested that the dysregulation of the TIFAB-USP15 complex, as seen in conditions like del(5q) myelodysplasia or MLL-rearranged leukemia, through the deubiquitination of MDM2 and KEAP1, may lead to a reduction in p53 expression. This, in turn, could enhance the activity of leukemic cells and contribute to the progression of leukemia [51].

Kuang and colleagues illustrated that by inhibiting USP1, they were able to reduce the levels of ID1 and p-AKT while triggering apoptosis in B-ALL cells. This suggests that USP1 promotes B-ALL progression through the ID1/AKT signaling pathway [52].

AlkB homolog 5 (ALKBH5) could be demethylated by N6 -methyladenosine (m6A) and this process has been found to promote cancer development in humans and enhance the stability of USP1 mRNA. When USP1 is overexpressed, it decreases the beneficial effects of an Aurora B inhibitor by interacting with Aurora B. Then it increase T‐ALL resistance to dexamethasone which could decrease cell apoptosis [53].

The activation of lymphocyte cell-specific protein-tyrosine kinase (LCK) activity results in a reduction in the expression of the glucocorticoid receptor (GCR) and leads to resistance against glucocorticoids. In their study, Jin and colleagues uncovered that USP11 collaborates with USP7 to remove ubiquitin molecules from the oncogenic protein LCK. This action boosts LCK’s activity, promoting the progression of an ALL while impairing normal blood cell formation and reducing sensitivity to GC [54].

E2A and HEB, which are E proteins, play a vital role in regulating the development of T cells as homodimers. When mice lack E2A or when inhibitors of E protein function like TAL1 or LYL1 are abnormally expressed in T cell precursors, it leads to the development of an aggressive disease similar to T-ALL [73]. This highlights that E proteins are essential for promoting the normal development of T cells while suppressing the formation of leukemia. In T-ALL, USP7 forms a co-regulatory complex with HEB, TAL1 and E2A then inhibiting T-ALL cell growth [55].

T-ALL mainly results from the abnormal activation of the NOTCH1 signaling pathway. Shan and their team illustrated that by reducing the degradation of NOTCH1, USP7 can mitigate apoptosis in T-ALL cells [56].

Tri-methylation of a specific site, lysine 27, on histone H3 (known as H3K27me3), is crucial for preventing the development of leukemia. During the initiation and progression of leukemia, there is a connection between the recruitment of NOTCH1 to target genes and the reduction of H3K27me3 levels, which occurs through the action of the enzyme Jumonji D3 (JMJD3). Additionally, a protein called USP7 plays a role by stabilizing NOTCH1 and subsequently activating the NOTCH1/JMJD3 pathway. This activation, in turn, increase the growth of T-ALL cells both in vivo and in vitro [57].

The NF-κB signaling pathway may become active due to the presence of oncoproteins in T-ALL leukemia cells [74]. In cases of T-ALL associated with human T cell leukemia virus type 1 (HTLV-1), the viral oncoprotein Tax plays a role in triggering leukemia through the activation of the NF-κB signaling pathway. USP20, by promoting the stability of TRAF6 and Tax, contributes to the activation of NF-κB, including its activation induced by IL-1β. Therefore, USP20 emerges as a potential target for the treatment of T-ALL [58].

Mcl-1, an antiapoptotic protein, is often overexpressed in tumor cells, which hinders the success of cancer therapy. Recent research suggests that BH3-only proteins like Bim, Puma, and Noxa can make Mcl-1 unstable. Trivigno et al. demonstrated that ionizing radiation activates USP9x, leading to increased deubiquitination of Mcl-1 in radioresistant T-ALL cells. This sensitizes them to apoptosis. However, their study found that BH3-only doesn’t regulate Mcl-1 stability or T-ALL cell survival upon irradiation. Additionally, irradiation doesn’t affect the protein levels of BH3-only proteins [59].

WP1130 (Bcr-abl inhibitor) has demonstrated significant effectiveness against various cancer types, although its potential for combating T-ALL remains uncertain. Luo and colleagues recently demonstrated that WP1130 treatment reduced the survival of T-ALL cells, prompting apoptosis, in vivo and in vitro. This effect was achieved by increasing the disruption of the mitochondrial transmembrane potential through the USP24-Mcl-1 pathway [60].

Takahashi and colleagues discovered that USP10 acts as an anti-stress factor in response to various environmental stressors, including viral infections and oxidative stress. When exposed to oxidative stress caused by arsenic, USP10 is mobilized into stress granules (SGs). These USP10-containing SGs help reduce the production of reactive oxygen species (ROS) and inhibit apoptosis triggered by ROS. However, the interaction between the Tax oncoprotein of HTLV-1 and USP10 has the opposite effect. It hinders the formation of SGs induced by arsenic, increases the production of ROS, and intensifies ROS-dependent apoptosis in T cells infected with HTLV-1. This interaction suggests that HTLV-1 Tax oncoprotein, when interacting with USP10, could potentially enhance apoptosis in T-ALL cells [61].

6TG is a long-standing medication currently employed in the treatment of leukemia, bowel disease, and rheumatoid arthritis. Research conducted by Chuang and colleagues indicates that based on enzyme kinetics and X-ray crystallography, 6TG operates as a noncompetitive inhibitor with a slow-binding mechanism against USP2. This research offers a solid justification for investigating the potential of 6TG as a treatment for cancers where USP2 activity is elevated [62].

Gavory and his team described reversible and specific compounds that inhibit USP7. These compounds operate through a clearly defined binding method and regulate the overall levels of substances downstream of USP7, such as MDM2, p21, and p53. This research demonstrated that inhibiting USP7 in ALL cells could lead to a reduction in MDM2 expression and an increase in p21 and p53 expression [63].

Microarray gene expression profiling is commonly used to study hematological malignancies. It has helped identify unique molecular signatures for different types of leukemia and has enabled the recognition of previously unknown subclasses within these diseases. De Pittà and coworkers’ study involved expression profiling of leukemia patients and successfully categorized them into three groups: MLL/AF4 translocation, B-ALL, and T-ALL which aligned with clinical classifications. The research identified 30 specific genes that effectively distinguished these subtypes. These 30 genes were validated using mini-array technology in a separate group of 17 patients. Additionally, the study uncovered two previously unreported genes, USP33 and endo mucin (EMCN), which were found to be overexpressed in B-ALL in comparison to their expression in T-ALL [64].

**Fig. 3:**
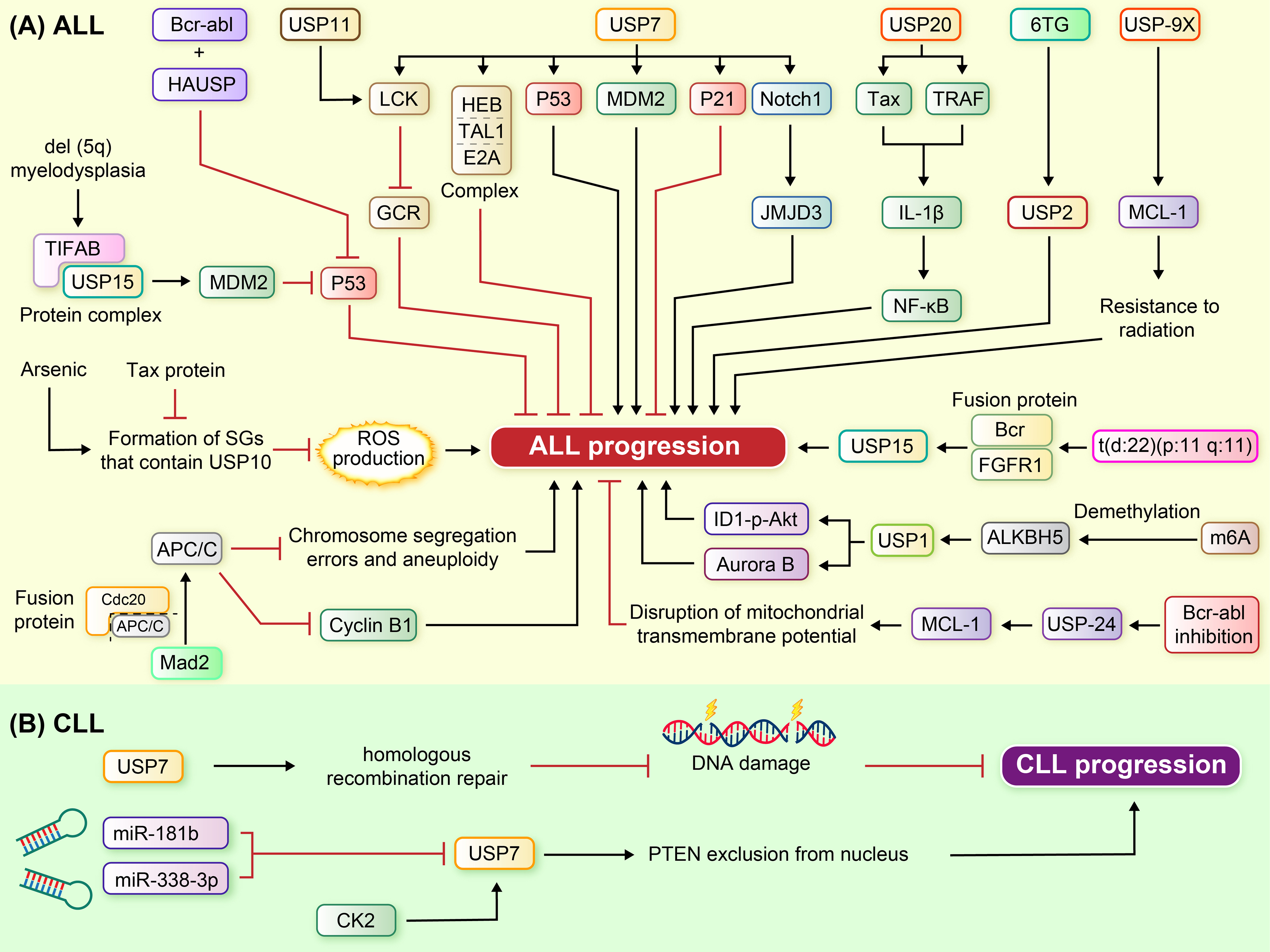
Signaling axis of the USP enzyme family that modulate lymphoid leukemia. (**A**) Specific fusion proteins (e.g., p190 BCR-ABL, BCR-FGFR1), splicing alterations, and dysregulation of ubiquitin-related pathways could impact ALL progression. Various deubiquitinase enzymes (e.g., USP7, USP15, USP1, USP10, USP9x, USP20, USP24) modulate key signaling pathways and proteins (e.g., p53, NOTCH1, Mcl-1) implicated in ALL pathogenesis. Potential therapeutic strategies, such as targeting USP2 with 6TG, inhibiting USP7, and using compounds like WP1130, enhance treatment efficacy in ALL. (**B**) Overexpression of USP7 in CLL cells disrupts HRR, causing DNA damage buildup and apoptosis, presenting a promising strategy for treating DDR-defective malignancies. MiR-181b, miR-338-3p, and CK2 influence USP7 levels, impacting CLL cell apoptosis and PTEN localization

The Cdc20-anaphase-promoting complex/cyclosome (Cdc20-APC/C) E3 ubiquitin ligase is crucial for proper mitotic progression and marking specific target proteins for degradation by the proteasome. USP44 was thought to suppress Cdc20-APC/C activity by keeping it associated with the inhibitory protein Mad2 until chromosomes are correctly attached to the mitotic spindle. Zhang et al. demonstrated that High Usp44 levels in murine embryonic fibroblasts promote Mad2-Cdc20 association and strengthen the mitotic checkpoint that results in chromosome segregation errors and aneuploidy and also could USP44 has an inhibitory effect on APC/C-Cdc20’s ubiquitination of cyclin B1 during the G2 phase. Also, they showed elevated USP44 expression is found in some T-ALL cells [65] (Fig. 3A).

### CLL

Chronic lymphocytic leukemia (CLL) is distinguished among malignancies by the accumulation of B-lymphocytes resistant to apoptosis. The persistence of CLL B-cells is attributed to factors like imbalances in cytoplasmic pro-survival and pro-death molecules such as Bcl-2, Mcl-1, and similar factors, influencing B-cell longevity. The molecular processes governing the onset and progression of CLL are not fully understood. However, recent advancements in next-generation sequencing have revealed new driver genes, some unexpected, providing insights into prognosis and potential therapeutic targets for CLL [75, 76].

In CLL the ATM-p53 pathway is often inactivated, leading to resistance to treatment and poor clinical outcomes. This study found that the protein USP7 is overexpressed in CLL cells. Inhibiting or removing USP7 disrupts homologous recombination repair (HRR), causing a significant buildup of DNA damage and leading to CLL cell apoptosis, even in cases where ATM and p53 are dysfunctional. Additionally, inhibiting USP7 makes p53-defective and chemotherapy-resistant CLL cells more responsive to clinically relevant doses of chemotherapy agents that induce HRR. So this research suggested that targeting USP7 could be a promising strategy for treating hematological malignancies with DDR defects, especially in cases where ATM and p53 cannot induce apoptosis [66].

Carrà and colleagues found that USP7 reduces apoptosis in CLL cells. Additionally, their study uncovered that miR-181b and miR-338-3p, by sponging USP7 mRNA, can reduce its protein levels. In contrast, CK2 can trigger PTEN’s exclusion from the cell nucleus by activating USP7 [67] (Fig. 3B).

### Future prospect

The future of leukemia management may involve modulating USP enzymes to better understand their roles in leukemia progression, suppression, and chemotherapy sensitivity. This could lead to personalized diagnostic and therapeutic strategies. Clarifying their roles in gene expression, protein stability, complex formation, histone deubiquitination, and protein repositioning in leukemia cells is crucial. Modulating USPs may provide insights into disease outcomes and improve the accuracy of leukemia subtype identification. Targeting USPs could enhance treatment efficacy and reduce side effects. Challenges include systemic cytotoxicity and off-target effects from non-specific UPS inhibition, impacting both healthy and malignant cells, and the intricate regulatory mechanisms governing ubiquitination that necessitate comprehensive understanding for precise targeting. Resistance observed with FDA-approved proteasome inhibitors highlights the need for novel therapeutic strategies to overcome resistance mechanisms. Clinical trials face challenges such as incomplete datasets due to funding constraints, hindering comprehensive evaluation. Developing potent and selective inhibitors or agonists for UPS enzymes that ensure effective tumor delivery while minimizing off-target effects remains a significant technical challenge. Future studies should focus on understanding ubiquitination codes, improving inhibitor specificity, securing clinical trial funding, developing targeted drug-delivery systems, and exploring combinatorial and synthetic lethality strategies [77]. Discovering new ubiquitination targets could expand therapeutic potential in leukemia and other diseases. Incorporating these strategies into personalized medicine and targeted therapies will enhance the precision and efficacy of leukemia treatments.

## Conclusion

USP enzymes represent a dynamic and promising area of research in leukemia. These enzymes play a crucial role in leukemia development by modulating key cellular processes. Understanding their involvement offers potential therapeutic targets to disrupt leukemia pathways and improve treatment. Furthermore, exploring the diagnostic and prognostic value of USP enzymes holds promise for personalized treatment. Continued research in this field has the potential to revolutionize leukemia management and provide hope for better outcomes in the future.

## Data Availability

The original contributions presented in the study are included in the article/supplementary material. Further inquiries can be directed to the corresponding author.

## Abbreviations

USPs: Ubiquitin-Specific Proteases
AML: Acute Myeloid Leukemia
CML: Chronic Myeloid Leukemia
ALL: Acute Lymphoblastic Leukemia
CLL: Chronic Lymphocytic Leukemia
UPS: Ubiquitin proteasome system
DUBs: Deubiquitinating enzymes
PTM: Post-translational modification
MPN: Myeloproliferative neoplasm
ITD: Internal tandem duplications
TG: Thioguanine
RNF220: Ring finger protein 220
DFS: Disease-free survival
OS: Overall survival
UCHL5: Ubiquitin C-terminal hydrolase 5
lncRNA: Long non-coding RNA
IFN: Type I interferon
ICD: Immunogenic cell death
ISGs: IFN-stimulated genes
RARA: Retinoic acid receptor alpha
PML: Promyelocytic leukemia
PLZF: Promyelocytic leukemia zinc finger
ATRA: All-trans retinoic acid
UBE1L: Ubiquitin-activating enzyme-E1-like
PML-NBs: PML-nuclear bodies
EBV: Epstein-Barr virus
NPC: Nasopharyngeal carcinoma
NPM1: Nucleophosmin
VOCs: Volatile organic compounds
TKIs: Tyrosine kinase inhibitors
SKP2: S-phase kinase-associated protein 2
MM: Multiple myeloma
ART: Artesunate
YBX1: Y-box-binding protein 1
FUS: Fused in sarcoma
DDR: DNA damage response
ID1: Inhibitor of DNA-binding-1
NiPT: Nickel pyrithione
hucMSC: Human umbilical cord mesenchymal stem cells
NGS: Next-generation sequencing
Ph: Philadelphia chromosome
HAUSP: Herpesvirus-associated ubiquitin-specific protease
FGFRs: Fibroblast Growth Factor Receptors
BCR: Breakpoint Cluster Region
SRSF: Serine/arginine-rich splicing factors
ALKBH5: AlkB homolog 5
m6A: N6 -methyladenosine
LCK: Lymphocyte cell-specific protein-tyrosine kinase
GCR: Glucocorticoid receptor
JMJD3: Jumonji D3 enzyme
HTLV-1: Human T cell leukemia virus type 1
SGs: Stress granules
ROS: Reactive oxygen species
EMCN: Endo mucin
Cdc20-APC/C: Cdc20-anaphase-promoting complex/cyclosome
HRR: Homologous recombination repair
