# Correction: Ubiquitin-specific proteases (USPs) in leukemia: a systematic review

**DOI:** 10.1186/s12885-024-12824-3

**Published:** 2024-09-02

**Authors:** Alireza Zangooie, Shima Tavoosi, Mahan Arabhosseini, Aram Halimi, Helia Zangooie, Amir Hossein Baghsheikhi, Soheila Rahgozar, Mohammad Ahmadvand, Alireza Mosavi Jarrahi, Zahra Salehi

**Affiliations:** 1 Student Research Committee, Birjand University of Medical Sciences, Birjand, Iran; 2 Cellular and Molecular Research Center, Birjand University of Medical Sciences, Birjand, Iran; 3 Department of Cell and Molecular Biology and Microbiology, Faculty of Biological Science and Technology, University of Isfahan, Isfahan, Iran; 4 Non-Communicable Diseases Research Center, Endocrinology and Metabolism Population Sciences Institute, Tehran University of Medical Sciences, Tehran, Iran; 5 Research Center for Social Determinants of Health, Research Institute for Endocrine Sciences, Shahid Beheshti University of Medical Sciences, Tehran, Iran; 6 Department of Epidemiology, School of Public Health and Safety, Shahid Beheshti University of Medical Sciences, Tehran, Iran; 7 Student Research Committee, Mashhad University of Medical Sciences, Mashhad, Iran; 8 Department of Biology, Science and Research Branch, Islamic Azad University, Tehran, Iran; 9 Cell Therapy and Hematopoietic Stem Cell Transplantation Research Center, Research Institute for Oncology, Hematology, and Cell Therapy, Tehran University of Medical Sciences, Tehran, Iran; 10 Cancer Research Centre, Shahid Beheshti University of Medical Sciences, Tehran, Iran; 11 Hematology, Oncology and Stem Cell Transplantation Research Center, Research Institute for Oncology, Hematology and Cell Therapy, Tehran University of Medical Sciences, Tehran, Iran

**Correction: BMC Cancer 24, 894 (2024)**

10.1186/s12885-024-12614-x

Following publication of the original article [1], the authors identified an error in the affiliations of Shima Tavoosi, Hossein Baghsheikhi, Soheila Rahgozar and Zahra Salehi. Shima Tavoosi is affiliated to institution 3, Hossein Baghsheikhi to institution 8, Soheila Rahgozar to institution 3, and Zahra Salehi is affiliated to institution 11.

The original article [1] has been corrected.
